# The Role of Cytokines which Signal through the Common γ Chain Cytokine Receptor in the Reversal of HIV Specific CD4^+^ and CD8^+^ T Cell Anergy

**DOI:** 10.1371/journal.pone.0000300

**Published:** 2007-03-21

**Authors:** Xiao Xiao Jenny Gu, Feng Yun Yue, Colin M. Kovacs, Mario A. Ostrowski

**Affiliations:** 1 Clinical Sciences Division, University of Toronto, Toronto, Canada; 2 St. Michael's Hospital, University of Toronto, Toronto, Canada; 3 Canadian Immunodeficiency Research Collaborative (CIRC), Toronto, Canada; University of California, San Francisco, United States of America

## Abstract

**Background:**

HIV specific T cells are putatively anergic *in vivo*. IL-2, a member of a class of cytokines that binds to receptors containing the common gamma chain (γc) has been shown to reverse anergy. We examined the role of γc cytokines in reversing HIV specific T cell anergy.

**Methods:**

PBMC from untreated HIV-infected individuals were briefly exposed to a panel of γc cytokines, and frequencies of *gag* specific T cells were enumerated by intracellular IFN-γ flow cytometry.

**Results:**

Of the γc cytokines, brief exposure to IL-2, IL-15, or combined IL-15/IL-7 significantly enhanced (range 2–7 fold) the CD4^+^ and CD8^+^ T cell IFN-γ responses to HIV *gag*, with IL-15 giving the greatest enhancement. The effects of cytokines were not due to enhanced proliferation of pre-existing antigen specific cells, but were due to a combination of enhanced cytokine production from antigen specific T cells plus activation of non-epitope specific T cells.

**Conclusions:**

These observations support the notion that a significant number of HIV specific T cells are circulating in an anergic state. IL-2, IL-7 and particularly IL-15 as an immune modulator to reverse HIV-1 specific T cell anergy should be investigated, with the caveat that non-specific activation of T cells may also be induced.

## Introduction

Although a potent CD4^+^ and CD8^+^ T cell immune response has been shown to control most virus infections, Human Immunodeficiency Virus (HIV) replication persists in the majority of infected individuals despite the presence of a detectable T cell immune response[1]–[5]. Analysis of T cell immune responses in HIV-infected individuals have revealed a number of apparent functional defects whose severity correlate closely with the degree of viral replication in the plasma. When compared to T cells of other viral specificities, HIV-specific T cells have been shown to have defects in proliferative capacity, cytokine production and effector function [6]–[14]. More specifically, using MHC-class I peptide-tetramer technology, HIV and SIV specific CD8^+^ T cells have been shown to be impaired in their ability to produce interferon-gamma (IFN-γ) in which less than 25% of tetramer-staining cells are able to produce cytokine in response to their cognate antigen [10], [11], [15], [16]. Similar defects have also been proposed in HIV-specific CD4^+^ T cells, in which, viral replication is associated with a defect in IL-2 production [7], [9], [17]. Since a direct comparison of HIV-specific CD4^+^ T cells by tetramer analysis and cytokine production is yet to be reported due to the difficulties in producing stable peptide-MHC class II tetramers, it is unknown what proportion of HIV-specific CD4^+^ T cells are defective in IL-2 or IFN-γ production in acute and chronic infection [18]. A number of mechanisms for this anergic or ‘stunned’ state of antigen specific T cells in HIV infection have been proposed, which include excessive activation from high antigen loads [7], [19], direct effects of gp120[20], or altered peptide ligands encoded due to viral mutants [21].

The common-gamma (γc) chain cytokines have been shown to be important growth factors for T cells [22] and of which IL-2, has been shown to reverse anergy *in vitro* [23], [24]. The common cytokine receptor gamma chain is essential for the function of at least six cytokines including IL-2, IL-4, IL-7, IL-9, IL-15, and IL-21. IL-2 is the most well characterized of these, and has been used in clinical trials to enhance CD4 counts in HIV infected individuals [25]–[28], however, its direct effect on HIV-specific T cells has not been well characterized. Although IL-2 is a T-cell growth factor, it also enhances apoptosis of mature T cells and induces tolerance by expanding CD25^+^ regulatory CD4^+^ T cells [29], [30]. IL-7 is important in T cell homeostasis by enhancing the survival of central memory T cells. IL-15 has been demonstrated to ensure the survival and proliferation of memory CD4^+^ and CD8^+^ T cells and NK cells. IL-4 primarily mediates the development of humoral immunity by promoting TH2 CD4^+^ T cells and B cell proliferation[22]. Thus, although members of the common γc cytokine family show considerable overlap, many differences in their functional characteristics are also found. In addition, it is unknown whether IL-7, IL-4 or IL-15 are capable of reversing anergy via signaling through the γc chain.

In the current study, we asked whether brief treatment of *ex vivo* HIV-specific T cells with various members of the γc cytokine family could reverse their anergic state. We sought to determine the relative effectiveness of these cytokines in reversing anergy as well as possible mechanisms as to how these cytokines work on antigen specific T cells.

## Materials and Methods

### Study participants

17 treatment naive HIV infected individuals were recruited for this study (see Table 1). Eight individuals were recently infected by HIV within 6 months of study (diagnosed by recent seroreactivity). Nine individuals with chronic HIV infection with disease progression were studied. This was defined as documented HIV infection >1 year, with evident CD4^+^ T cell decline of >50 CD4 cells/ mm^3^ and a viral load >10,000 copies/ml. All investigational protocols were approved by the University of Toronto and St. Michael's hospital Institutional Review Boards.

**Table 1:** Profiles of HIV-1 Infected Participants

Patient	Clinical Diagnosis	CD4 Count/mm3	Viral Load(copies/ml)
1	Recent Sero	660	440,000
2	Recent Sero	810	224,000
3	Recent Sero	180	28,650
4	Recent Sero	300	150,660
5	Recent Sero	440	8,571
6	Recent Sero	550	250,000
7	Recent Sero	600	118,778
8	Recent Sero	363	171,830
9	Chronic	132	22,415
10	Chronic	410	40,021
11	Chronic	539	175,000
12	Chronic	170	47,000
13	Chronic	200	75,000
14	Chronic	80	50,964
15	Chronic	520	10,719
16	Chronic	340	200,000
17	Chronic	200	105,497

Recent Sero-Recently acquired HIV-1 infection within 6 months

Chronic HIV-HIV-1-infected >1 year with CD4 decline

### Source of antigens

Forty-nine overlapping 20mer peptides spanning the *gag* region of HIV-IIIB (amino acids 1–500), overlapping by 10 amino acids, were obtained from the NIH AIDS Research Reagent program (catalog # 3992), dissolved in DMSO and used as a pool with each peptide at a final concentration of 1.0 µg/ml. DMSO in appropriate dilutions was used as a negative control.

### Cell preparation

Peripheral blood mononuclear cells (PBMC) were Ficoll-separated (LSM, Organon Teknika, Durham, NC) from freshly obtained blood samples and washed in PBS (BioWhittaker, Walkersville, MD) and cultured in RPMI-10%HAB serum (BioWhittaker, Walkersville, MD). Cells were cultured overnight (16 hours) in the following conditions, a) medium alone, b) IL-2 (25U/ml), c) IL-15 (25 ng/ml), d) IL-7 (25 ng/ml), e) IL-15+IL-7 (each at 25 ng/ml), and f) IL-4 (25ng/ml). IL-2 was obtained from the NIH AIDS Reagent Program whereas IL-15, Il-7 and IL-4 were obtained from R&D Systems (Minneapolis, MN). The following day, cells from each condition were washed of cytokines with PBS×2 and then resuspended in RPMI-10%HAB medium and stimulated for 12 hours by an HIV *gag* peptide pool (see above) or control antigen (DMSO) in the presence of monensin and 1 µg/ml of anti-CD49d and CD-28 antibodies for co-stimulation (BD Biosciences, San Diego, CA). Cells were then harvested and assessed for intracellular cytokines.

### Flow cytometry and intracellular cytokine determination

The procedure for intracellular staining of cytokines in PBMCs was performed using the Cytofix/cytoperm Plus kit according to the manufacturer's instructions (Cat# 554715, BD Biosciences, San Diego, CA). Cells were washed, fixed and permeabilized in FACS permeabilization buffer and were stained by a panel of conjugated antibodies (fluorescein isothiocyanate (FITC), phycoerythrin (PE), peridinin chlorophyll protein (PerCP) and allophycocyanin (APC) including antibodies to human CD4, CD8, CD69 (Pharmingen), and IFN-γ and respective isotype controls. Cells were then washed and resuspended in 1% paraformaldehyde/PBS and then analyzed the following day on a FACSCalibur (BD Biosciences, San Diego, CA). All antibodies were obtained from BD Biosciences. In selected experiments, PBMC from HLA-A *0201 individuals who had CD8^+^ T cell responses to the SLYNTVATL epitope of HIV *gag* were also stained with the MHC-I tetramer to this peptide (iTag, Beckman-Coulter, Fullerton, CA). In the latter experiments cells were washed three times with PBS to ensure removal of peptide prior to tetramer staining. Data were acquired by Cell Quest software (BD Biosciences, San Diego, CA) and analyzed using FloJo (Treestar Inc., San Carlos, CA). From 100,000 to 200,000 events in the lymphocyte gate were acquired/sample.

### Proliferation assays

To more specifically determine the effect of cytokines on the proliferative activity of antigen specific cells in our assays, stable incorporation of the intracellular fluorecent dye 5-(and–6)-carboxyfluorescein diacetate succinimidyl ester (CFSE) was employed. PBMCs were suspended in PBS containing 5% FBS and were stained at room temperature for 5 min with 5 µM CFSE. Staining was terminated by adding PBS containing 5% fetal bovine serum (FBS) and subsequent washing with PBS. Cells were then treated similarly as above for antigen specific assays. Proliferation was assessed by measuring dilution of CFSE staining on antigen specific cells that were co-stained with antibodies to CD4, CD8 and IFN-γ.

### Statistical analysis

Data were compared using the Wilcoxon signed rank test for paired samples (two-tailed).

## Results

### Effect of brief exposure to γc cytokines on HIV specific T cell responses

**Figure 1:**
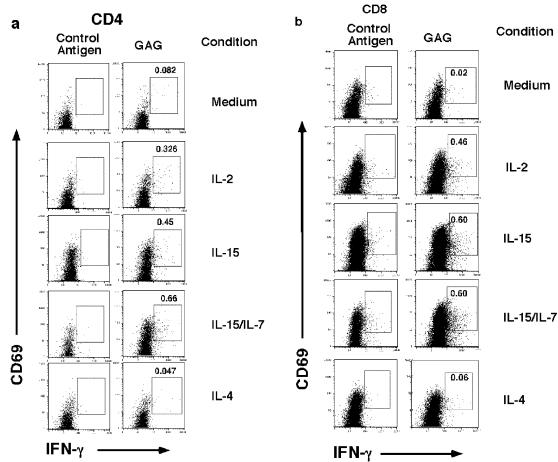
*Effect of γc cytokines on HIV specific T cell responses in a chronic progressor.* Fresh *ex vivo* PBMC from an HIV infected individual with chronic progression (Pt# 11) were incubated overnight in the following culture conditions: medium, IL-2 (20U/ml), IL-15 (20 ng/ml), IL-15+IL-7 (20 ng/ml each), or IL-4 (20 ng/ml). The following day, cells were washed twice, resuspended in plain medium and then stimulated with control antigen (DMSO) or a *gag* peptide pool in the presence of Monensin, and then stained for intracellular cytokines. Shown are dot plots of *gag* or control antigen stimulated samples from PBMC in respective culture conditions, for CD4^+^ T cells (a) or CD8^+^ T cells (b). The upper right numbers in the right hand panels indicate the % of HIV *gag* specific IFN-γ producing CD4^+^ or CD8^+^ T cells after subtraction from DMSO controls (left panel).

**Figure 2:**
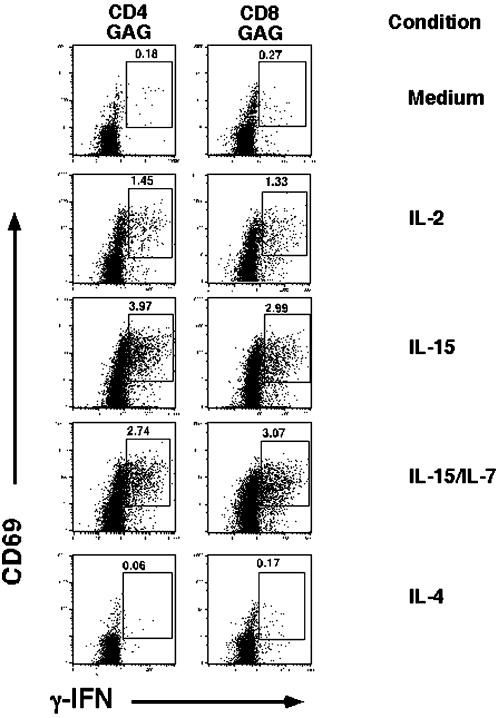
*Effect of γc cytokines on HIV specific T cell responses in an acute seroconverter.* Fresh *ex vivo* PBMC from an HIV infected individual with acute HIV infection (Pt# 5) were incubated overnight similarly to as in Figure 1. Shown are dot plots of HIV *gag* antigen stimulated samples for CD4^+^ T cells and CD8^+^ T cells (DMSO control conditions not shown). The upper right numbers indicate the % of HIV *gag* specific IFN-γ producing CD4^+^ (left panel) or CD8^+^ T cells (right panel) after subtraction from DMSO controls (not shown).

Since *gag* is the most conserved protein of HIV and elicits the most frequent T cell responses in HIV infected individuals, we elected to study the effects of γc cytokines on the T cell response to HIV *gag* in a cohort of untreated HIV infected individuals with recent seroconversion or chronic progressive disease (see Table 1). Individuals with recent seroconversion were studied because of the high levels of immune activation and viremia associated with this stage of infection [31]–[34]. We asked whether short term overnight treatment of HIV- *gag* specific T cells could reverse any defect in IFN-γ production that may be occurring *in vivo*. Freshly obtained *ex vivo* PBMC were treated overnight in plain medium or with the following cytokines or combination, thereof: IL-2, IL-15, IL-4 or IL-7+IL-15. The latter combination of IL-7+IL-15 was previously shown to have a synergistic effect on inducing the proliferation of memory T cells [22]. The following day, PBMC were washed extensively to remove residual cytokines, exposed to HIV *gag* or control antigen, and then assessed for their ability to produce IFN-γ as measured by intracellular flow cytometry. An example of a representative experiment from Participant #11, a chronic progressor, is shown in Figure 1a and b depicting both control and antigen stimulated conditions in CD4^+^ (Fig. 1a) and CD8^+^ T (Fig. 1b) cells. The frequency of HIV *gag* specific T cells was calculated by measuring IFN-γ/CD69 expressing cells observed in HIV *gag* containing conditions and subtracting from DMSO (control antigen) containing conditions, similar to as previously described [35]. In this individual, when PBMC were cultured in plain medium the measured frequency of *gag* specific IFN-γ producing CD4^+^ T was .082%. However, if the PBMCs were briefly exposed to IL-2, the measured frequency increased to 0.326%, if they were exposed to IL-15, the frequency was 0.45%, to both IL-15/IL7 it was 0.66%, whereas, if cells were briefly exposed to IL-4, we only detected a frequency of 0.047%. Similarly, for CD8^+^ T cells, we detected a 0.02% frequency of *gag* specific IFN-γ producing cells if PBMC were briefly cultured in plain medium, whereas the frequencies increased to 0.46%, 0.6%, 0.6% or 0.06% if they were briefly cultured in the presence of IL-2, IL-15, IL-15/IL-7, or IL-4, respectively. Data from participant #5, a recent seroconverter is also depicted in Figure 2. When combining data from all 17 individuals (Table 2), the frequency of *gag* specific CD4^+^ T cells detected after brief treatment with medium, IL-2, IL-15, IL-15/7 or IL-4 were 0.08%, 0.23% (p<0.005 vs medium), 0.50% (p<0.001 vs medium), 0.53% (p<0.005 vs medium) and 0.08% (p = n.s vs medium) respectively, and for *gag* specific CD8^+^ T cells; 0.52%, 1.0% (p<0.001 vs medium), 1.58% (p<0.001 vs medium), 2.0% (p<0.001 vs medium) and 0.52% (p = n.s vs medium), respectively. In addition, the frequency of *gag* specific CD4^+^ and CD8^+^ T cells was significantly greater with IL-15 or IL-15/7 compared to IL-2 (p<.05, and p<.005 respectively). Although, treating CD4^+^ and CD8^+^ T cells with IL-15/7 combined tended to give higher responses compared to IL-15 alone, the differences failed to reach statistical significance. Culturing cells in the presence of IL-4 failed to enhance *gag* specific T cell responses. Thus, when *ex vivo* PBMC are cultured overnight in the presence of the γc cytokines, IL-2, IL-15, or combined IL-15/IL-7 one can enhance the CD4^+^ and CD8^+^ T cell IFN-γ responses to HIV *gag*.

**Table 2:** Summary data from 17 HIV-1 infected individuals with chronic progressive or acute HIV-1 infection.

Condition (overnight incubation)	medium	IL-2	IL-15	IL-15+IL-7	IL-4
Mean frequency of gag specific CD4 (% of CD4+ T cells)	0.08	0.23#	0.50*	0.53#	0.08̂
Mean frequency of gag specific CD8(% of CD8+ T cells)	0.52	1.00*	1.58*	2.00*	0.52̂

* p<0.001 compared to medium; #p<0.005 compared to medium; ^p = not significant compared to medium

### Effect of brief exposure of γc cytokines on proliferation of HIV specific cells

**Figure 3:**
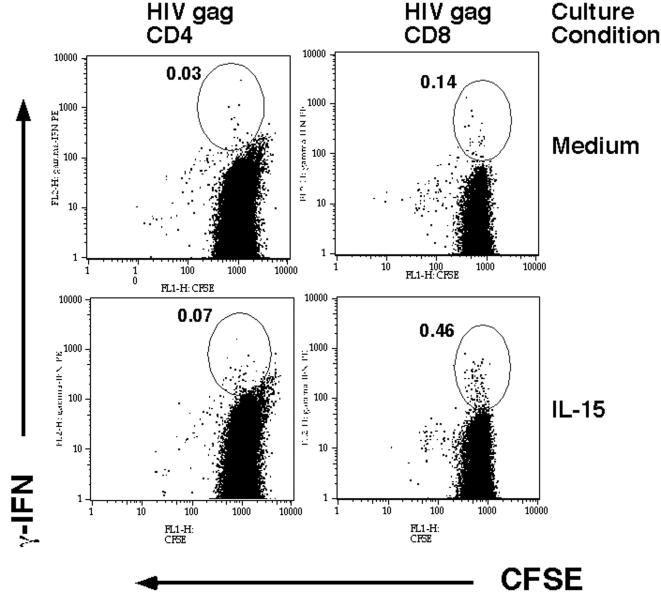
*Effect of γc cytokine treatment on proliferation of HIV specific T cells.* Ex vivo PBMC obtained from Participant #7 were labeled with CFSE and then incubated in medium or IL-15 as described above. PBMC were then washed and then exposed to HIV *gag* or control antigen (not shown) and tested for intracellular IFN-γ. Shown are dot plots of antigen stimulated conditions for CD4^+^ and CD8^+^ T cells. The increased numbers of IFN-γ producing T cells after IL-15 exposure is not associated with any dilution of CFSE staining. Numbers represent % of HIV *gag* specific IFN-γ producing cells. Shown, are representative data of one of three experiments.

We saw a dramatic enhancement (range 2–7 fold) in the frequency of IFN-γ producing T cell responses after exposure to IL-2 or IL-15. One possibility is that exposure to these cytokines allowed proliferation of antigen specific cells overnight *in vitro* prior to their detection by intracellular cytokine staining. In order to address this, in selected participants, PBMC were stained with CFSE prior to performing experiments in order to assess proliferation of antigen specific cells via the effect of proliferation on diluting the CFSE stain. A representative experiment comparing medium and IL-15 conditions in Participant #7 is shown in Figure 3. We find that IFN-γ producing T cells do not dilute CFSE in the presence of IL-15 indicating that the enhanced frequency of IFN-γ producing cells is not due to proliferation overnight.

### Antigen specificity of enhanced responses due to γc cytokines

**Figure 4:**
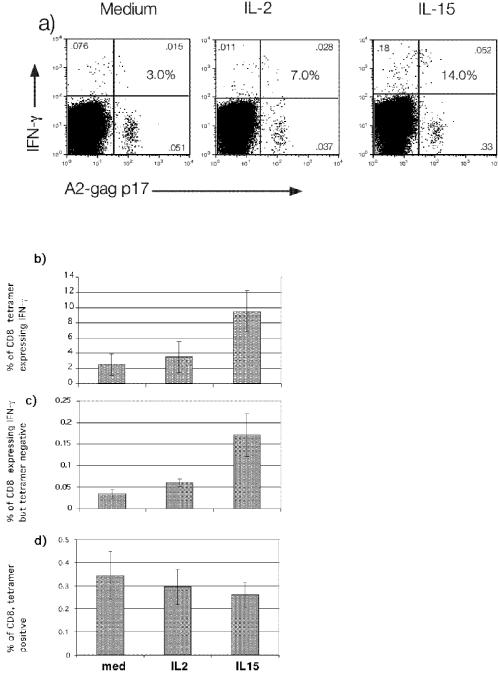
*Effect of γc cytokines on p17 specific CD8^+^ T cells*. PBMC from three HLA A*0201 individuals with chronic progression were cultured overnight in medium, IL-2 or IL-15 as described in Figure 1. Cells were then washed and exposed to the A*0201 restricted p17 peptide of HIV and then stained for intracellular IFN-γ, CD8, and with a p17 specific tetramer. Dots plots for one individual are shown in (a) after gaiting on CD8. Large numbers (%) in upper right quadrants indicate the% of tetramer staining cells that co-stain for IFN-γ. Small numbers in all quadrants represent % of total CD8^+^ T cells. Summary data from all three individuals are shown depicting in (b) the % of tetramer staining cells which also produce IFN-γ (upper R/upper R+lower R quadrant) , in (c) the % of CD8^+^ T cells expressing IFN-γ but do not stain for tetramer (upper L quadrant/ total CD8s), and in (d) the number of tetramer staining CD8^+^ T cells in all conditions (upper and lower R quadrant/ total CD8s). Bar graphs depict means and bars represent standard errors on the mean.

An alternative possibility of the enhanced responses observed with IL-2 and IL-15 pretreatment may be related to activation of cells not specific for the antigen. In order to address this, we examined the effect of overnight IL-2 and IL-15 treatment on CD8^+^ T cells specific for the HLA-A2*0201 restricted epitope of HIV *gag*, SLYNTAVTL, using tetramer co-staining experiments. PBMC from three HLA-A2*0201 positive individuals with chronic progressive HIV infection, who previously showed detectable IFN-γ responses to the this epitope, were treated overnight with medium, IL-2, or IL-15, and then were stimulated for 6 hours with the SLYNTVATL peptide, and assessed for intracellular IFN-γ production by flow cytometry. A representative experiment is depicted in Figure 4a, with summary data from all three individuals shown in Figures 4b-d. As demonstrated with the CFSE experiments, brief cytokine treatment did not enhance the proliferation of antigen specific T cells since the numbers of tetramer staining cells were similar with all three conditions (Figure 4a and d). SLYNTAVTL specific CD8^+^ T cells from these three individuals showed severe anergy, as <10% of tetramer staining cells could produce IFN-γ upon stimulation. Brief treatment with IL-2 or IL-15 was able to partially reverse this anergy, with IL-15 being more effective than IL-2 at increasing the proportion of tetramer staining cells to produce IFN-γ (Figure 4a and b). The detection of increased IFN-γ expressing cells after SLYNTVATL stimulation was however, also associated with an increase frequency of tetramer negative but IFN-γ positive cells, which was most significantly seen in the IL-15 containing condition, indicating that a portion of the IL-15 effect is not SLYNTVATL specific (Figure 4a and c). Since a portion of the enhancing effect of IL-2 and in particular, IL-15 was due to cells that were not antigen specific, we looked at the expression of the activation marker, CD69, in cells that were pretreated with γc cytokines in the absence of HIV antigen. As can be observed in Figure 1a and b, baseline CD69 expression is enhanced in control antigen conditions in cells pretreated with IL-2 or IL-15. For all samples, the baseline levels of CD69 expression on CD4+ T cells with medium, IL-2, IL-15, IL-15/7, and IL-4 were 2.3%, 5.0%, 7.5%, 5.9% and 2.9% respectively, (p<.05 for medium vs IL-2, IL-15 and IL-15/7, data not shown); and on CD8+ T cells with medium, IL-2, IL-15, IL-15/7, and IL-4 were 4.1%, 7.8%, 17.7%, 15.1% and 4.7%, respectively, (p<.05 for medium vs IL-2, IL-15 and IL-15/7, data not shown). Thus, brief pretreatment of T cells with γc cytokines IL-2, and IL-15 but not IL-4 enhances their activation state, which is more pronounced with IL-15.

## Discussion

In the current study, we show that brief treatment of *ex vivo* PBMCs obtained from untreated HIV infected individuals, with the γc cytokines IL-2, IL-15 or IL-15 plus IL-7 can to varying extents, enhance the ability of HIV-specific CD4^+^ and CD8^+^ T cells to produce IFN-γ. Our findings support those of others in which the addition of IL-15 could enhance IFN-γ ELISpot responses to CMV, or PPD in healthy donors[36], to HIV in treated HIV-infected children[37], and to SIV in SIV infected macaques[38]. These observations support the notion that a significant number of HIV-1 specific CD4^+^ and CD8^+^ T cells are circulating in an anergic or unresponsive state. This was confirmed by finding that γc cytokines increase the proportion of HIV specific CD8^+^ T cells directed against the SLYNTVATL epitope (p17) to produce IFN-γ, and that this effect occurred independantly of any effect on proliferation of these cells. IL-15 or the combination of IL-15 plus IL-7 was the most potent at reversing anergy, whereas, IL-4 had no effect. Although IL-15 could enhance the ability of p17-specific CD8^+^ T cells to produce IFN-γ, the majority of tetramer positive cells still could not secrete IFN-γ in response to peptide stimulation, indicating the intense anergic state of these cells in the individuals studied. It is possible that more prolonged culture of cells (e.g., 1–2 weeks) in the presence of cytokines may have more extensively reversed this defect. We used a short (12 hour) exposure in our study in order to avoid any effect of these cytokines on proliferation of antigen specific cells *in vitro*, and because we were interested in determining whether *ex vivo* antigen specific cells were indeed anergic.

The lack of significant effects with IL-4 suggests that signaling through the common γc alone is not sufficient to reverse anergy but that signaling through additional cytokine specific chains like IL-2Rβ and IL-15β may also be necessary. All of the γc cytokines, activate the jak1 and jak3 kinases upon binding to their specific and the common gamma chains, respectively. However, downstream to jak activation, IL-2, IL-15 and IL-7 signaling then specifically phosphorylate stat 3 and stat 5, whereas, IL-4 signaling phosphorylates stat 6. Thus, it appears likely that stat 3 and stat 5 activation are responsible in part for the effects observed. The mechanisms as to how IL-2 and IL-15 enhance the effect of signaling through the TCR are beginning to emerge. Recently Liu et al.[39] showed by genetic profiling microarray analysis, that 73% of genes upregulated by IL-15 on memory T cells overlap with genes upregulated by TCR signals on the same cells. These findings suggest that signals induced through the TCR could be amplified via IL-15 as similar genes are being upregulated, with the resulting effect being decreasing the threshold for T cell activation through the TCR after exposure to antigen. This is consistent with what we observed in our cohort, as, IL-2, IL-15 and IL-15/7 enhanced the baseline activation state of T cells by enhancing CD69 expression, a marker of activation through the TCR. Thus, the beneficial effect of IL-2 or IL-15 appeared to occur at the expense of enhanced background activation of T cells. In our tetramer analysis, the effects of enhanced activation was associated with enhanced IFN-γ production from cells which did not appear to be specific to the peptide stimulus. It is unclear whether this represents activation of cells with TCRs that cross-react to the peptide, and thus are activated because the threshold of activation was lowered, or reflect completely non-specific activation.

The γc cytokines, including IL-2, IL-15 and IL-7 are currently receiving consideration for treatment in HIV infection as systemic therapies. Our data suggests that all should potentially be useful in enhancing the functional capacity of HIV specific T cells *in vivo*, with IL-15 or combined IL-15 plus IL-7 showing the most potent effects. Clinical use of these cytokines however may also be associated with an enhanced activation state of T cells, particularly with IL-15, which may have both positive effects or negative consequences. A positive effect would be the recruitment of potentially cross reactive T cells that could deal with virus variants that escape from the immunodominant response. The negative effect would be that enhanced immune activation would facilitate further HIV viral replication.
